# Using machine learning to predict rapid decline of kidney function in sickle cell anemia

**DOI:** 10.1002/jha2.168

**Published:** 2021-02-10

**Authors:** Fatma Güntürkün, Daiqing Chen, Oguz Akbilgic, Robert L. Davis, Ibrahim Karabayir, Maxwell Strome, Yang Dai, Santosh L. Saraf, Kenneth I. Ataga

**Affiliations:** ^1^ Center for Biomedical Informatics University of Tennessee Health Science Center Memphis USA; ^2^ Department of Bioengineering University of Illinois at Chicago Chicago USA; ^3^ Department of Health Informatics and Data Science Loyola University Chicago Maywood USA; ^4^ Department of Econometrics Kirklareli University Kirklareli Turkey; ^5^ Department of Computer Science University of Michigan Ann Arbor USA; ^6^ Department of Medicine University of Illinois at Chicago Chicago USA; ^7^ Center for Sickle Cell Disease University of Tennessee Health Science Center Memphis USA

**Keywords:** chronic kidney disease, machine learning models, predictive capacity, rapid decline of eGFR, sickle cell disease

Chronic kidney disease (CKD) is prevalent in sickle cell disease (SCD) [1]. Kidney function declines more rapidly in SCD than in the general African‐American population [2]. Furthermore, rapid decline in kidney function, defined as an estimated glomerular filtration rate (eGFR) loss of >3 mL/min/1.73 m^2^ per year [3, 4], occurs more commonly than in the general population [5, 6]. As rapid kidney function decline is associated with increased mortality in SCD [2], early identification of patients at risk for such decline may facilitate risk modification.

Machine learning (ML), characterized as the study of algorithms and statistical models that computer systems utilize to learn from previous experience, can assess relationships of multiple variables, create predictions based on characteristics and identify patient groups with comparable patterns [7]. We explored the potential of ML tools to predict rapid kidney function decline in SCD, hypothesizing that ML models are highly predictive of rapid kidney function decline in severe SCD genotypes.

Participants in this retrospective cohort study have been previously described [2, 5]. The internal cohort consisted of SCD patients, ≥18 years old, seen during routine clinic visits from 2004–2013 (Figure S1). Only patients with two or more measures of kidney function over the observation period were evaluated. The external cohort consisted of patients, ≥18 years old, followed from 2009–2020. Institutional Review Board approvals were obtained at the University of North Carolina at Chapel Hill and the University of Illinois at Chicago.

Data collected included demographics, clinical laboratory tests, SCD‐related complications, co‐morbid conditions and treatments. At each visit, GFR was estimated using the creatinine‐based CKD Epidemiology Collaboration (CKD‐EPI) formula [8]. Visits in which rapid eGFR decline occurred were defined using two thresholds: (a) eGFR loss > 3.0 mL/min/1.73 m^2^ per year and (b) eGFR loss > 5.0 mL/min/1.73 m^2^ per year [3, 4]. Data were censored at the first occurrence of rapid eGFR decline. As the first occurrence of rapid decline may reflect acute kidney injury (AKI), additional analyses were performed, restricted to patients with at least two visits in 1 year and persistent or sustained decline in kidney function. Analyses were performed for each eGFR decline threshold and for prediction of rapid decline 6 and 12 months following a clinic visit. Missing data imputation, predictive modeling, and sensitivity analysis are shown in Figure S2/Supplementary Data. Serum creatinine, hemoglobin, reticulocyte count, history of stroke, diabetes, avascular necrosis, hydroxycarbamide therapy, systolic blood pressure (SBP), diastolic blood pressure, use of ACE‐inhibitors/angiotensin receptor blockers, weight, age, sex, eGFR, and prior eGFR slope were all used as predictors for each model.

Two hundred and thirty‐six patients (HbSS–218, HbSβ^0^–18), mean age 31.15 (±11.9) years, 133 (56.4%) females, with a mean observation period of 4.9 (±3.1) years were evaluated in the internal cohort. Rapid kidney function decline occurred in 166 (70.3%) patients based on first visit with eGFR decline >3 mL/min/1.73 m^2^ per year, and in 140 (59.3%) patients based on first visit with eGFR decline >5 mL/min/1.73 m^2^ per year. Baseline characteristics of the cohorts, stratified by rapid eGFR decline status, are shown in Table 1. In the external cohort, 168 patients (HbSS), mean age of 32.5 (±9.8) years, 86 (51.2%) females, with mean observation period of 7.2 (±2.18) years were evaluated. Rapid eGFR decline based on a threshold of >3 mL/min/1.73 m^2^ per year was observed in 145 (86.3%) patients, while eGFR decline >5 mL/min/1.73 m^2^ per year was seen in 136 (80.9%) patients.

**TABLE 1:** Baseline demographic, laboratory, and clinical characteristics of internal and external cohorts with stratification for rapid decline status

	Internal cohort (mean [±SD]/number [%])		External cohort (mean [±SD]/number [%])	
Covariates	No rapid decline at any visit (N = 70)	Rapid decline at >3 mL/min/1.73 m^2^ threshold (N = 166)	No rapid decline at any visit (N = 23)	Rapid decline at > 3 mL/min/1.73 m^2^ threshold (N = 145)
Age (years)	29.0 (9.66)	32.06 (12.64)	31.88 (6.40)	32.58 (10.26)
Weight (kg)	68.23 (15.9)	68.27 (16.58)	72.27 (12.35)	70.21 (14.27)
Serum creatinine (mg/dL)	0.74 (0.26)	0.78 (0.39)	0.83 (0.41)	0.74 (0.50)
Hemoglobin (g/dL)	9.01 (1.67)	8.90 (1.55)	9.21 (1.33)	8.92 (1.78)
Hemoglobin F (%)	8.99 (6.32)	7.85 (6.88)	7.17 (5.36)	8.73 (5.86)
Blood urea nitrogen (mg/dL)	9.07 (4.74)	9.92 (8.23)	7.96 (4.92)	8.06(6.95)
Absolute reticulocyte count (x10^9^/L)	238.38 (106.2)	234.83 (122.78)	295.3 (173.34)	324.6 (154.09)
Systolic blood pressure (mm Hg)	121.23 (15.88)	119.81 (15.60)	131.3 (24.76)	120.1 (18.71)
Diastolic blood pressure (mm Hg)	71.04 (10.51)	69.77 (14.81)	77.26 (17.92)	70.51 (12.16)
White blood cell count (10^9^/L)	11.25 (3.89)	10.01 (3.53)	9.37 (2.59)	10.11 (3.94)
Total bilirubin (mg/dL)	3.69 (2.93)	2.99 (2.21)	2.72 (1.21)	3.38 (2.57)
Estimated glomerular filtration rate (mL/min/1.73m^2^)	135.32 (25.94)	132.88 (37.60)	128.70 (36.68)	136.22 (30.84)
Sex (female)	46 (65.71)	87 (52.41)	10 (43.5)	76 (52.41)
Proteinuria (yes)	15 (35.71)	33 (37.08)	2 (8.69)	41 (28.28)
Hemoglobinuria (yes)	7 (17.5)	14 (17.72)	4 (17.39)	47 (32.41)
Hydroxyurea use (yes)	29 (41.43)	69 (42.07)	16 (69.6)	82 (56.55)
ACE Inhibitor/ARB therapy (yes)	6 (8.7)	18 (10.84)	1 (4.3)	25 (17.24)
Chronic RBC transfusion (yes)	2 (2.86)	9 (5.42)	0	9 (6.21)
History of diabetes (yes)	2 (2.86)	5 (3.01)	0	3 (2)
History of stroke (yes)	9 (14.06)	27 (17.76)	2 (8.7)	27 (18.62)
History of avascular necrosis (yes)	17 (34.69)	47 (41.59)	6 (26)	44 (30.3)
History of acute chest syndrome (yes)	57 (85.07)	140 (88.05)	14 (60.87)	83 (54.24)
History of leg ulcers (yes)	9 (16.07)	29 (20.57)	3 (13.04)	21 (14.48)

*ACE inhibitor/ARB therapy, angiotensin converting enzyme inhibitor/angiotensin receptor blocker therapy.

Estimated GFR decline >3 mL/min/1.73 m^2^ at 6 months was predicted with 82% sensitivity, 80% accuracy, and AUC of 0.88 (95% confidence interval [CI]: 0.79–0.97), while eGFR decline > 5 mL/min/1.73 m^2^ at 6 months was predicted with 79% sensitivity, 84% accuracy, and AUC of 0.91 (95% CI: 0.85–0.97). Estimated GFR decline at 12 months was predicted with 53% sensitivity, 70% accuracy, and AUC of 0.67 (95% CI: 0.53–0.81) with the >3 mL/min/1.73 m^2^ threshold, and with 64% sensitivity, 65% accuracy, and AUC of 0.77 (95% CI: 0.67–0.87) at >5 mL/min/1.73 m^2^ threshold (Figures S3 and S4).

In subjects with at least two consecutive eGFR slopes  >3 mL/min/1.73 m^2^, in the model predicting decline in 6 months, the AUC was 0.82 (95% CI: 0.72–0.91), with 78% sensitivity, and 78% accuracy. For the >5 mL/min/1.73 m^2^ threshold, the AUC was 0.87 (95% CI: 0.78–0.95), with 77% sensitivity and 81% accuracy. For the model predicting eGFR decline at 12 months, at the >3 mL/min/1.73 m^2^ threshold, AUC was 0.60 (95% CI: 0.47–0.72), with 45% sensitivity, and 67% accuracy, and at the >5 mL/min/1.73 m^2^ threshold, AUC remained unchanged, with 63% sensitivity, and 65% accuracy (Table 2). In the external cohort, prediction of eGFR decline >3 mL/min/1.73 m^2^ at 6 months was similar to that of the internal cohort, while prediction of eGFR decline >5 mL/min/1.73 m^2^ 6 months in advance was poorer than in the internal cohort (Table 2).

**TABLE 2:** Performance of the prediction models in internal and external cohorts*

		eGFR decline threshold of >3 mL/min/1.73 m^2^					eGFR decline threshold of > 5 mL/min/1.73 m^2^ (cases and controls/cases and controls with eGFR decline <3 mL/min/1.73 m^2^)**				
		Accuracy	Recall	Precision	F1	AUC	Accuracy	Recall	Precision	F1	AUC
Internal Cohort	6 months in Advance	0.80	0.82	0.82	0.81	0.88	0.84	0.79	0.81	0.79	0.91
	12 months in Advance	0.70	0.53	0.61	0.54	0.67	0.65	0.64	0.41	0.43	0.77
External Cohort	6 months in Advance	0.82	0.84	0.93	0.88	0.82	0.67/0.75	0.66/0.75	0.81/0.93	0.72/0.83	0.73/0.85
	12 months in Advance	0.63	0.65	0.82	0.72	0.71	0.61/0.63	0.61/0.62	0.65/0.80	0.63/0.70	0.61/0.64

* Random forest was used for the >5 mL/min/1.73 m^2^ threshold and 12‐month prediction window; AdaBoost was used for >3 mL/min/1.73 m^2^ threshold and other prediction windows.

** Performance of the models at the >5 mL/min/1.73 m^2^ threshold was evaluated in two different ways: (a) all cases and controls; (b) all cases and controls who had an eGFR slope >−3. Controls with eGFR declines between −3 and −5 mL/min/1.73 m^2^ were excluded.

In evaluation of feature importance, age, baseline eGFR, eGFR slope 6 months previously, reticulocyte count and SBP were associated with >3 mL/min/1.73 m^2^ eGFR decline threshold at 6 months and had similar importance overall (Figure S5). Age and eGFR slope 6 months previous were the most strongly predictive of rapid eGFR decline at >5 mL/min/1.73 m^2^ threshold. Baseline eGFR, age, SBP, serum creatinine and eGFR slope in the previous 12 months predicted rapid eGFR decline for both >3 mL/min/1.73 m^2^ and >5 mL/min/1.73 m^2^ thresholds at 12 months.

Rapid eGFR decline at 6 months was predicted with fairly high accuracy using ML models, but less so at 12 months. Despite concerns that the first decline in eGFR might reflect AKI, predictive capacities based on sensitivity analyses were only minimally decreased suggesting that use of the first eGFR decline reflected rapid kidney function decline. Notwithstanding the suitability of ML methods, predictions of distant events may not always be possible as evident by the modest predictive capacities of our models at 12 months. Biomarkers of kidney injury and larger patient populations may be necessary to better predict long‐term outcomes.

Our study differs from prior studies evaluating kidney function decline in that we calculated rate of eGFR decline at each visit, while others have calculated decline over the entire observation period [5, 9]. We have also used eGFR calculated at each visit to predict future decline. Furthermore, in our model development, we used all the data up to any point in time to predict eGFR decline 6 and 12 months in advance.

Despite limitation by lack of quantification of albuminuria, use of real‐world data, and some eGFR data imputation, this study demonstrates a role for ML models to predict rapid decline in eGFR. With the association of rapid eGFR decline with mortality in SCD, ML may play an important role in identifying patients at high risk for progressive kidney disease as early as 6 months in advance. More studies are required to further evaluate ML models in SCD‐related kidney disease.

## CONFLICT OF INTEREST

The authors declare that there is no conflict of interest that could be perceived as prejudicing the impartiality of the research reported. Dr. Ataga has received funding from the US FDA (R01‐FD006030), Novartis, and Global Blood Therapeutics, served on advisory boards for Novartis, Global Blood Therapeutics, Novo Nordisk, Editas Medicine, Forma Therapeutics and Agios Pharmaceuticals, and as a consultant for Roche. Dr. Saraf receives research funding support from Novartis, Pfizer, and Global Blood Therapeutics and served on advisory boards for Novartis and Global Blood Therapeutics. The project described was supported in part by the National Institutes of Health through grants R03HL146788 (Santosh L. Saraf) and R01HL153161 (Santosh L. Saraf).

## AUTHOR CONTRIBUTIONS

Fatma Güntürkün analyzed the data and wrote the manuscript. Daiqing Chen analyzed the data and wrote the manuscript. Oguz Akbilgic analyzed the data and assisted with manuscript preparation. Robert L. Davis analyzed the data and wrote the manuscript. Ibrahim Karabayir analyzed the data and assisted in the manuscript preparation. Maxwell Strome analyzed the data and assisted with manuscript preparation. Yang Dai analyzed the data and assisted with manuscript preparation. Santosh L. Saraf assisted in study design and manuscript preparation. Kenneth I. Ataga designed the study and wrote the manuscript.

## Supporting information

Supporting Information
