# Supplementary material for: Using machine learning to predict rapid decline of kidney function in sickle cell anemia
Source: EJHaem. 2021 Feb 10;2(2):257–60. doi: 10.1002/jha2.168 (PMC9176130; doi:10.1002/jha2.168)

**SUPPLEMENTARY DATA**

**Statistical Methods**

***Missing Data Imputation****:* As rapid decline was evaluated at each visit, rather than at the last visit, data were imputed for those visits where such variables were unavailable. Data were imputed with the multivariate imputation by chained equations (MICE) method, using the MICE package in R. MICE method imputes missing values for covariates using a chained equation process. MICE method offers various approaches for the chained equation process including predictive mean matching (PMM), classification and regression trees (CART), random forest (RF), and sample. Multivariate imputation techniques work well with up to 50% missingness rates. As such, we limited our use of predictors to covariates with less than 50% missingness. Using creatinine values, we identified the optimal method for imputation, one with the least error, to be PMM, also a preferred method for skewed data (Table 1). We imputed continuous covariates with PMM and categorical covariates with logistic regression.

Serum creatinine was imputed for 25% of visits in the internal cohort and for 49% of visits in the external cohort

***eGFR Decline Rate****:* At each visit, linear regression was used on eGFR data from current and prior visits to calculate the rate of eGFR change preceding that visit. The first visit with yearly regression slope lower than -3 or -5 mL/min/1.73 m^2^ was labeled as rapid eGFR decline. After identifying the first visit with rapid eGFR decline, we analyzed covariates from visits six months and 12 months before and treated them as predictors. We also analyzed covariates six months and 12 months before the final visit in those patients without rapid eGFR decline (controls). Patients who did not have a visit six (±1.8) months or 12 (±3) months before the first rapid eGFR decline were excluded.

***Predictive Modeling****:* We used logistic regression and decision tree-based ML algorithms, including CART, and four ensemble methods including RF, adaptive boosting (AdaBoost), gradient boosting (GB) and extreme gradient boosting (XGBoost) to predict occurrence of rapid eGFR decline at six months and 12 months. Model performance was evaluated by applying five-fold cross validation and a variety of performance metrics, including overall classification accuracy, recall, precision, F1-score and area under the receiver operating characteristic curve (AUC). After constructing predictive models, using scikit-learn Python library, a feature importance analysis was performed to identify covariates with the most impact on rapid eGFR decline.

***Sensitivity Analysis****:* With concerns that the first occurrence of rapid decline may reflect acute kidney injury (AKI), additional analyses were performed, restricted to patients who had at least two visits in one year with either persistent or sustained decline in kidney function. After a first decline, eGFRs may continue to decrease, return to its previous level, or may have intermittent stepwise changes that then remain stable over time (Supplementary Figure 2). Subjects who did not have a second visit within one year following the first eGFR decline were excluded.

**SUPPLEMENTARY TABLES**

**Table 1: Performance Results of Multivariate Imputation by Chained Equations (MICE) in Each Method**

| **Method** | **Mean Absolute Error** | **Mean Square Error** | **Root Mean Square Error** | **Mean Absolute Percentage Error** |
| --- | --- | --- | --- | --- |
| CART | 0.11 | 0.08 | 0.29 | 0.10 |
| RF | 0.13 | 0.05 | 0.23 | 0.13 |
| PMM | 0.09 | 0.03 | 0.18 | 0.10 |
| Sample | 0.45 | 0.52 | 0.72 | 0.54 |

* CART - Classification and Regression Trees; RF - Random Forest; PMM - Predictive Mean Matching

**Table 2: Performance of Machine Learning Algorithms in Predicting Rapid eGFR Decline in Internal Cohort**

|  | | **eGFR Decline**  **Threshold of >3 mL/min/1.73 m^2^** | | | | | **eGFR Decline**  **Threshold of >5 mL/min/1.73 m^2^** | | | | |
| --- | --- | --- | --- | --- | --- | --- | --- | --- | --- | --- | --- |
|  |  | **Accuracy** | **Recall** | **Precision** | **F1** | **AUC** | **Accuracy** | **Recall** | **Precision** | **F1** | **AUC** |
| **6 Months in Advance** | Logistic Regression | 0.42 | 0.53 | 0.43 | 0.47 | 0.41 | 0.46 | 0.34 | 0.34 | 0.33 | 0.45 |
|  | Decision Tree | 0.73 | 0.70 | 0.80 | 0.73 | 0.74 | 0.77 | 0.70 | 0.77 | 0.72 | 0.78 |
|  | Random Forest | 0.79 | 0.87 | 0.77 | 0.82 | 0.84 | 0.82 | 0.79 | 0.77 | 0.76 | 0.83 |
|  | AdaBoost | 0.80 | 0.82 | 0.82 | 0.81 | 0.88 | 0.84 | 0.79 | 0.81 | 0.79 | 0.91 |
|  | Gradient Boosting | 0.80 | 0.84 | 0.82 | 0.82 | 0.86 | 0.80 | 0.73 | 0.78 | 0.74 | 0.87 |
|  | XGBoost | 0.82 | 0.84 | 0.83 | 0.83 | 0.87 | 0.83 | 0.74 | 0.86 | 0.79 | 0.85 |
| **12 Months in Advance** | Logistic Regression | 0.56 | 0.34 | 0.39 | 0.35 | 0.50 | 0.66 | 0.51 | 0.38 | 0.43 | 0.58 |
|  | Decision Tree | 0.62 | 0.51 | 0.59 | 0.51 | 0.63 | 0.74 | 0.46 | 0.65 | 0.39 | 0.67 |
|  | Random Forest | 0.67 | 0.40 | 0.71 | 0.46 | 0.63 | 0.65 | 0.64 | 0.41 | 0.43 | 0.77 |
|  | AdaBoost | 0.70 | 0.53 | 0.61 | 0.54 | 0.67 | 0.72 | 0.63 | 0.49 | 0.50 | 0.74 |
|  | Gradient Boosting | 0.67 | 0.49 | 0.58 | 0.51 | 0.64 | 0.71 | 0.63 | 0.51 | 0.55 | 0.76 |
|  | XGBoost | 0.65 | 0.25 | 0.67 | 0.35 | 0.52 | 0.64 | 0.55 | 0.39 | 0.38 | 0.72 |

**SUPPLEMENTARY FIGURES**

**FIGURE LEGENDS**

**Figure 1: Project Workflow.** The analysis steps of the project: determining the visits where the first eGFR decline occurred based on 2 different thresholds, developing models that will predict these declines 6 months and 12 months in advance, validating the models with the highest performance in external data.

**Figure 2: Different eGFR trajectories after the first rapid eGFR decline: a) stable decrease in eGFR; b) further decrease in eGFR; and c) return of eGFR to its previous level**. Following determination of the visit where the first eGFR decline occurred based on yearly regression slope, we plotted the different trajectories in eGFR.

**Figure 3: ROC Curve Analysis of Classifiers at 6 Months: a) Threshold of >3 ml/min/1.73 m^2^; b) Threshold of >5 ml/min/1.73 m^2^**. Receiver operating characteristic curves for prediction of rapid eGFR decline based on thresholds of >3 ml/min/1.73 m^2^ and >5 ml/min/1.73 m^2^ comparing six machine learning models.

**Figure 4: ROC Curve Analysis of Classifiers at 12 Months: a) Threshold of >3 ml/min/1.73 m^2^; b) Threshold of >5 ml/min/1.73 m^2^**. Receiver operating characteristic curves for prediction of rapid eGFR decline based on thresholds of >3 ml/min/1.73 m^2^ and >5 ml/min/1.73 m^2^ comparing six machine learning models.

**Figure 5: Feature Importance Analyses: a) at 6 Months; b) at 12 Months.** Features with the highest contribution to the detection of eGFR decline at 6 months and 12 months, respectively.

**Supplementary Figure 1: Project Workflow**

Imputation of missing data

eGFR calculated at time of each visit

Threshold 1 Threshold 2

eGFR loss >3.0 mL/min/1.73 m^2^ eGFR loss >5.0 mL/min/1.73 m^2^

Identify date of first eGFR decline Identify date of first eGFR decline

**Retrospective cohort**

4131 clinic visits for 236 patients with severe SCD genotypes (HbSS/HbSβ^0^) at a single medical center from 2004 - 2013

**Data preprocessing**

- Logistic Regression - Decision Tree (CART) - Adaptive Boosting

- Random Forest - Gradient Boosting - Extreme Gradient Boosting

Model to predict eGFR decline

6 months in advance 12 months in advance

**Modeling to predict first eGFR decline**

External validation of prediction models in a cohort including 13870 clinic visits of 168 patients with HbSS/HbSβ^0^ at a single medical center

**External validation**

**Supplementary Figure 2: Different eGFR trajectories after the first rapid eGFR decline**

a) Stable decrease in eGFR


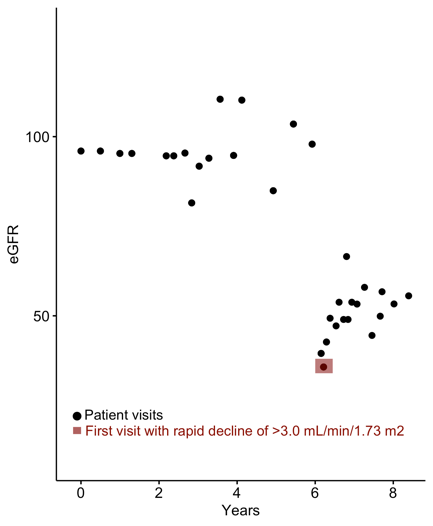


b) Further decrease in eGFR
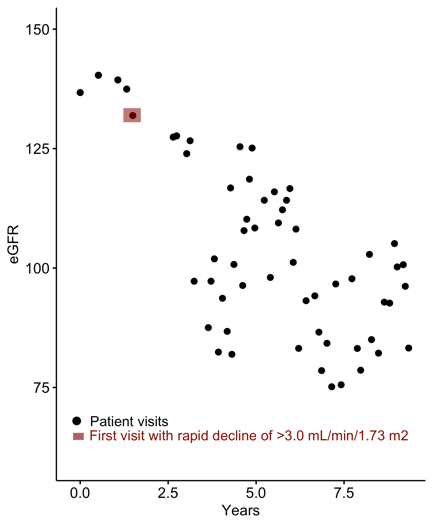
c) Return of eGFR to its previous level


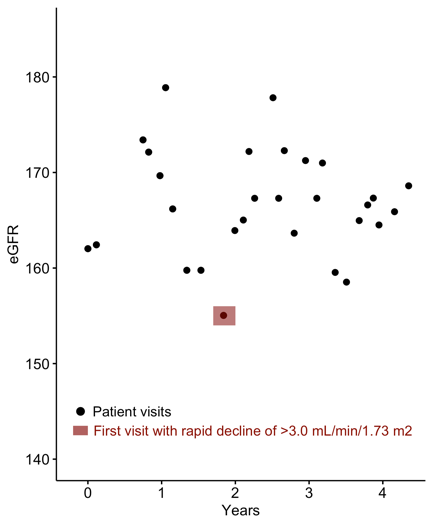


**Supplementary Figure 3: ROC Curve Analysis of Classifiers at 6 months**

**a) Threshold of >3 mL/min/1.73 m^2^ b) Threshold of >5 mL/min/1.73 m^2^**


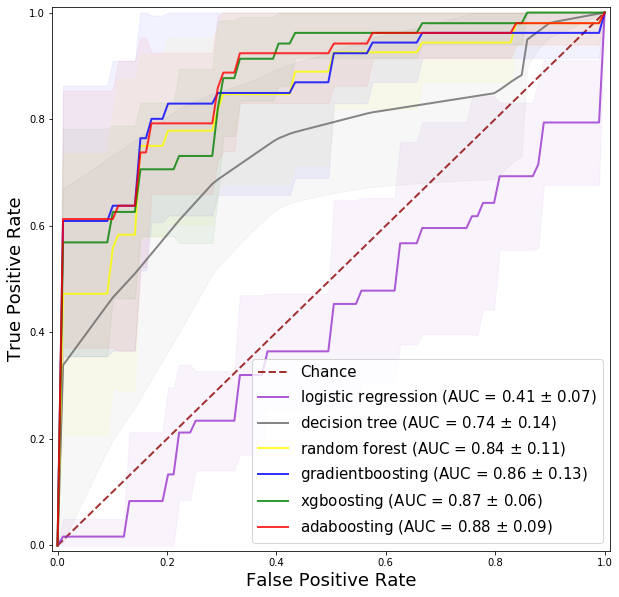


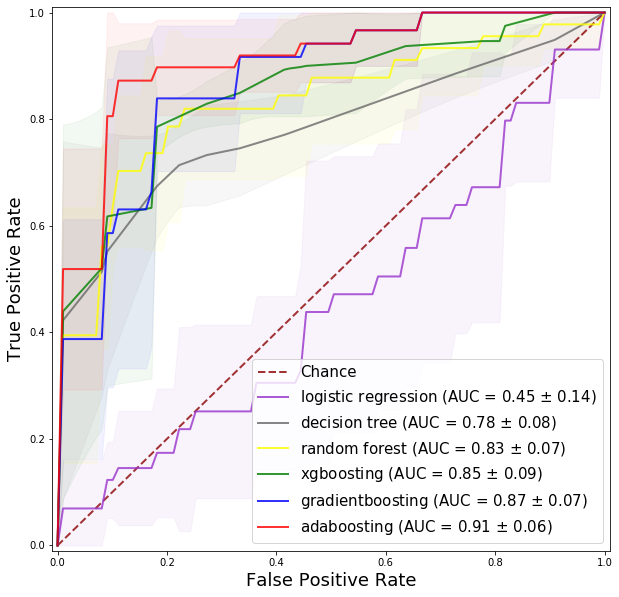


**Supplementary Figure 4: ROC Curve Analysis of Classifiers at 12 months**

**a) Threshold of >3 mL/min/1.73 m^2^ b) Threshold of >5 mL/min/1.73 m^2^**


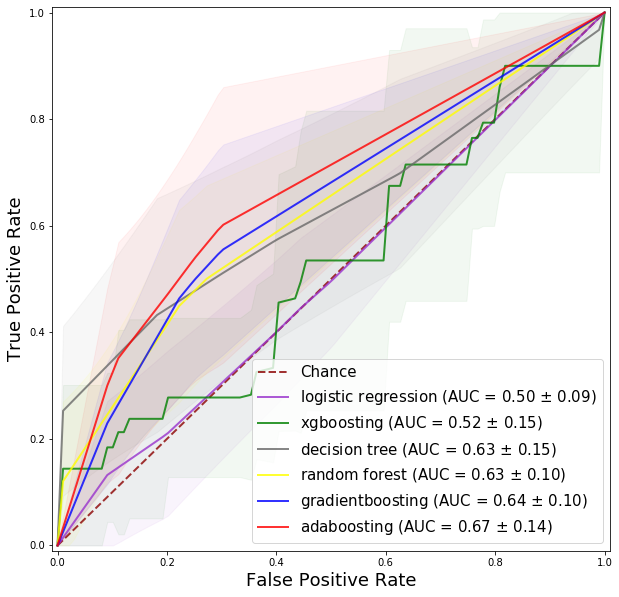


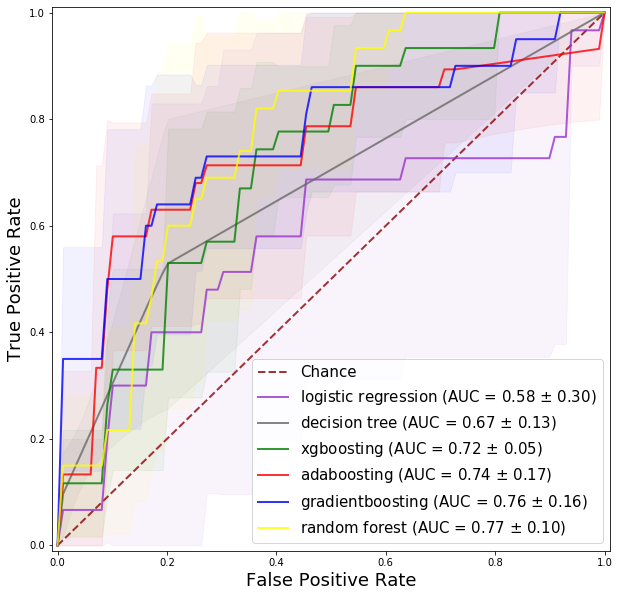


**Supplementary Figure 5: Feature Importance Analyses**

**a) at 6 months b) at 12 months**


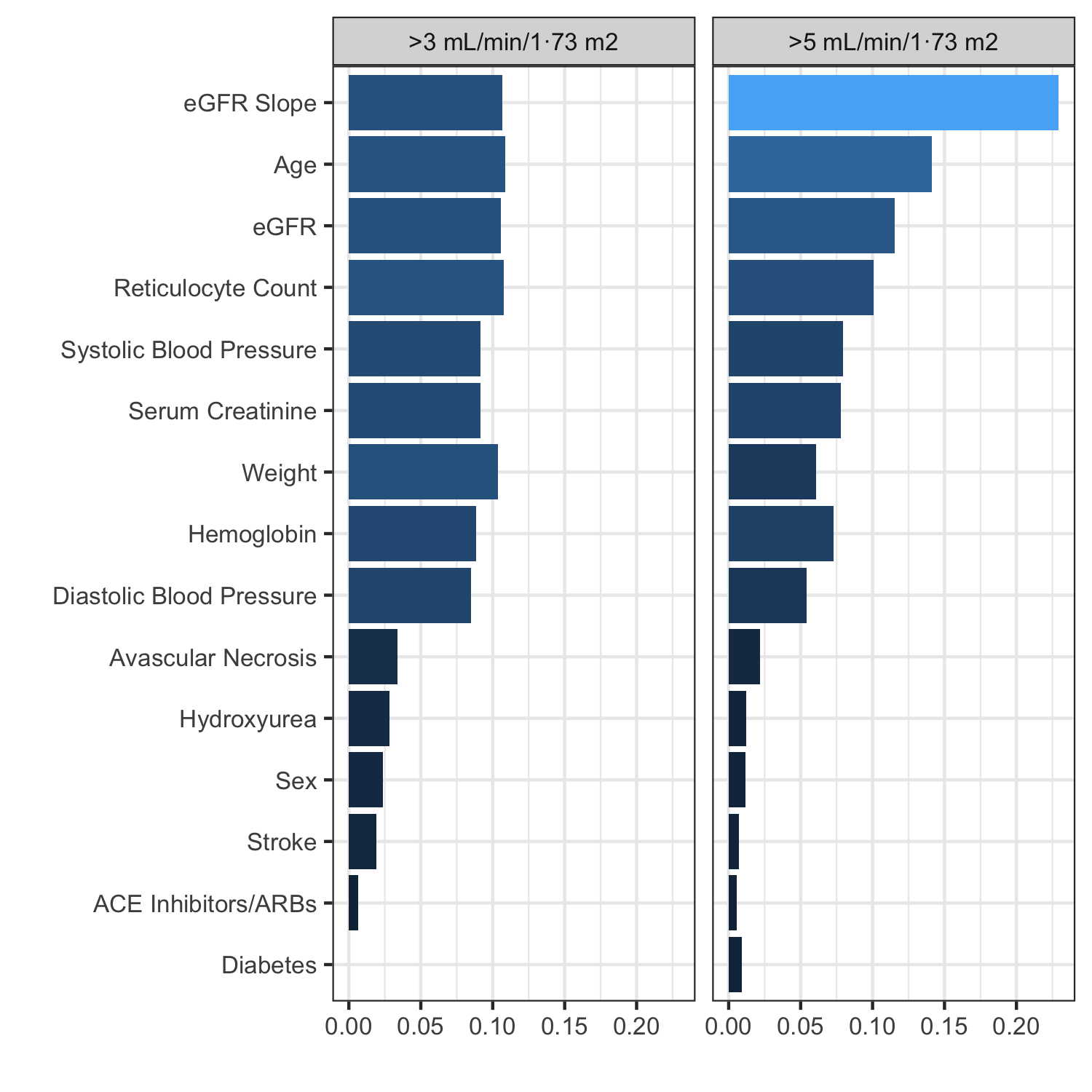


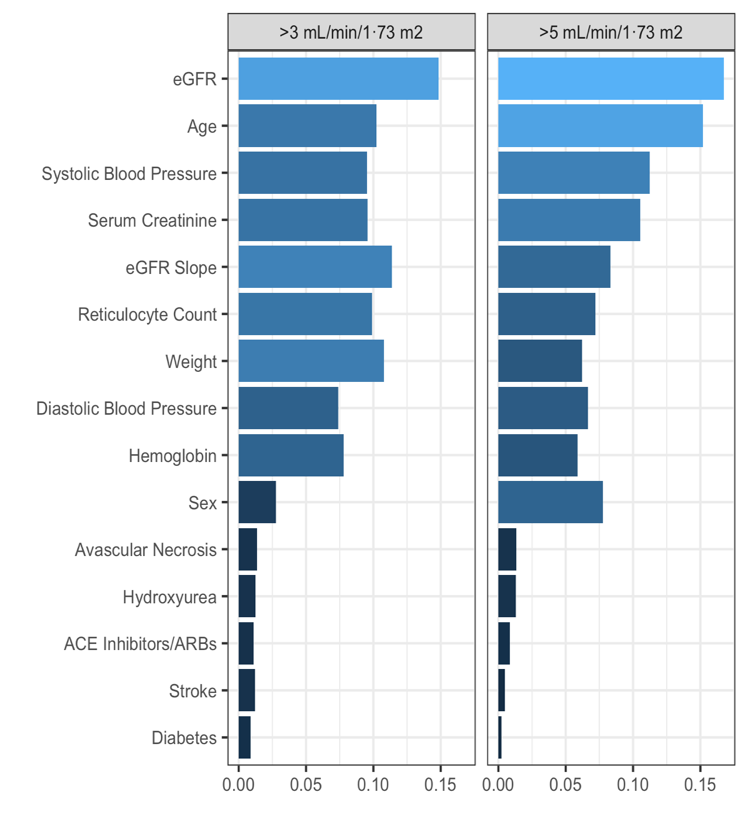

Supplement: Supplementary file 1 — Supporting Information [file JHA2-2-257-s001.docx]
